# Comparison of survival benefits of nephron-sparing intervention or active surveillance for patients with localized renal masses: a systematic review and meta-analysis

**DOI:** 10.1186/s12894-019-0503-3

**Published:** 2019-08-05

**Authors:** Run-Qi Guo, Xiao-Guang Li

**Affiliations:** Minimally Invasive Tumor Therapies Center, Beijing Hospital, National Center of Gerontology, No.1 Dongdan Dahua Street, Beijing, 100370 People’s Republic of China

**Keywords:** Ablation, Active surveillance, Renal mass, Partial nephrectomy, Survival

## Abstract

**Background:**

Strong evidence comparing effectiveness between nephron-sparing intervention (NSI) and active surveillance (AS) is lacking. Thus, we aim to compare the outcomes of survival, including cancer-specific survival (CSS), overall survival (OS), and cardiovascular-specific survival (CVSS), in patients with renal masses who underwent NSI or AS.

**Methods:**

A systematic literature search of PubMed, Web of Science, and EMBASE was performed for citations published prior to September 2018 that described NSI, partial nephrectomy and thermal ablation included, and AS for patients with renal masses and a standard meta-analysis on survival outcomes was then conducted.

**Results:**

The meta-analysis included seven studies containing 5809 patients. The results comparing NSI with AS were as follows: CSS (hazard ratio (HR) = 0.64, 95% confidence interval (CI): 0.46–0.89, *P* < 0.001), OS (HR = 0.46, 95%CI: 0.39–0.53, *P* < 0.001), and CVSS (HR = 0.37, 95%CI: 0.24–0.57, *P* < 0.001).

**Conclusions:**

This systematic review and meta-analysis indicates that NSI is associated with better OS, CSS and CVSS when compared with AS for patients with renal masses. Further better prospective cohort studies are needed to make definitive statements about these different treatment methods.

## Background

Renal masses range from benign tumors to cancers that can be indolent or aggressive, of which 80–90% are renal cell carcinoma (RCC) [1, 2]. RCC represents 2–3% of all cancers, with the highest incidence occurring in Western countries [3, 4]. Surgical resection, including radical nephrectomy and partial nephrectomy (PN), remains an effective treatment for clinically localized RCC. Given a low RCC-specific mortality for the elderly and those with small renal masses and other competing-cause mortality [5], active surveillance (AS) and thermal ablation (TA) such as cryoablation or radiofrequency ablation are alternative options for selected patients. Cardiovascular comorbidity and survival is especially relevant for RCC patients [6–8]. Thus, these approaches are being progressively supported by professional organizations, especially for patients with extensive comorbidities or decreased life expectancy. Each of the management options has its own relative risks and advantages in different patients, and no strict criteria for patient selection have been advocated by current guidelines. Furthermore, strong evidence comparing effectiveness between nephron-sparing intervention (NSI) (PN and TA included) and AS is lacking.

Given the need to clarify existing management algorithms for renal masses, the objective of present study was to compare the outcomes of survival, including cancer-specific survival (CSS), overall survival (OS), and cardiovascular-specific survival (CVSS), in patients with renal masses undergoing NSI or AS.

## Methods

### Search strategy

A bibliographic search was from January 2000 to September 2018 on PubMed, EMBASE and Web of Science. Institutional Review Board approval was not required as this study was evidenced-based review. All the records are written in English. The main key words used for the search were “ablation” or “cryoablation” or “radiofrequency ablation” or “microwave ablation” or “partial nephrectomy”, “active surveillance” or “watchful waiting” or “expectant management”, “prognosis” or “survival” or “oncological outcome”, and “kidney cancer” or “renal tumor”. Besides, we reviewed the reference lists from the related articles manually. Only full-text articles published in peer-reviewed journals were identified.

### Eligibility criteria

The identified studies were screened in accordance with the following criteria: (1) studies comparing the effectiveness or survival between NSI (enucleation, PN, and TA, like radiofrequency ablation, cryoablation, microwave ablation, etc.) and AS; (2) studies that clearly described outcome assessment by representing it in OS or CSS or CVSS; (3) survival outcome further demonstrated hazard ratio (HR) and corresponding 95% confidence interval (CI) or adequate data to achieve an estimated HR and 95% CI by using the methods reported by Tierney et al [9]; (4) prospective cohort or retrospective study; and (5) median follow-up of at least 12 months.

The exclusion criteria were as follow: (1) The literature was review, letter, case reports and meta-analysis; (2) The data were not available; (3) The literature deal with recurrent RCC, metastatic carcinoma, or urothelial carcinoma; and (4) Duplicate publication.

Two researchers identified all the publications that fit the criteria for the assessment of the titles and abstracts and full review. Divergences were settled through discussion.

### Data extraction

The two reviewers extracted data from full length articles independently. The extracted data included the followings: name of the author, publication year, study region, study period, study design, sample size, intervention, median age, median follow-up duration, number of patients received intervention or active surveillance, median tumor size, outcomes including CSS or OS or CVSS. Any differences were settled by consensus.

### Quality assessment

The quality of each study was determined using the modified Newcastle-Ottawa Scale [10]. This scale evaluates the risk in three aspects: selection of patients, comparability of NSI and AS groups and evaluation of the treatment outcome. Studies with scores less than 4 were considered to have a high risk of bias, scores of 4–6 to have a moderate risk of bias, and scores over 6 to have a low risk of bias. Each study was evaluated by two researchers independently. Disagreement was reassessed by both the researchers until consensus was reached.

### Data analysis and synthesis

Log HR and the variance were utilized as the summary outcome measure from all studies in the meta-analysis. For each study, HR with the 95%CI of the survival rate was derived to compare the effectiveness between NSI and AS for patients with renal masses. Each meta-analysis was repeated after excluding Surveillance, Epidemiology, and End Results (SEER) data. Fixed-effects model, namely Mantel-Haenszel method, was applied to pool the results when there was no evident heterogeneity (*I*^*2*^ > 50% and *P* value < 0.1 suggested obvious heterogeneity) [11], otherwise, the random-effects model (DerSimonian and Laird method) was utilized, which provided more conservative estimates when there was significant heterogeneity [12]. Meta-regression analysis and subgroup analysis were conducted for potential sources of inter-study heterogeneity.

The funnel plot was constructed for each meta-analysis to detect the publication bias, and the Egger’s test was utilized to assess publication bias statistically [13]. All analyses were performed using Review manager version 5.3 (Cochrane Collaboration, Oxford, UK) and STATA version 14.0 (State Corporation, College Station, TX, USA). A *P* value was regarded as statistical significance when less than 0.05.

## Results

### Literature search and characteristics of studies

**Fig. 1:**
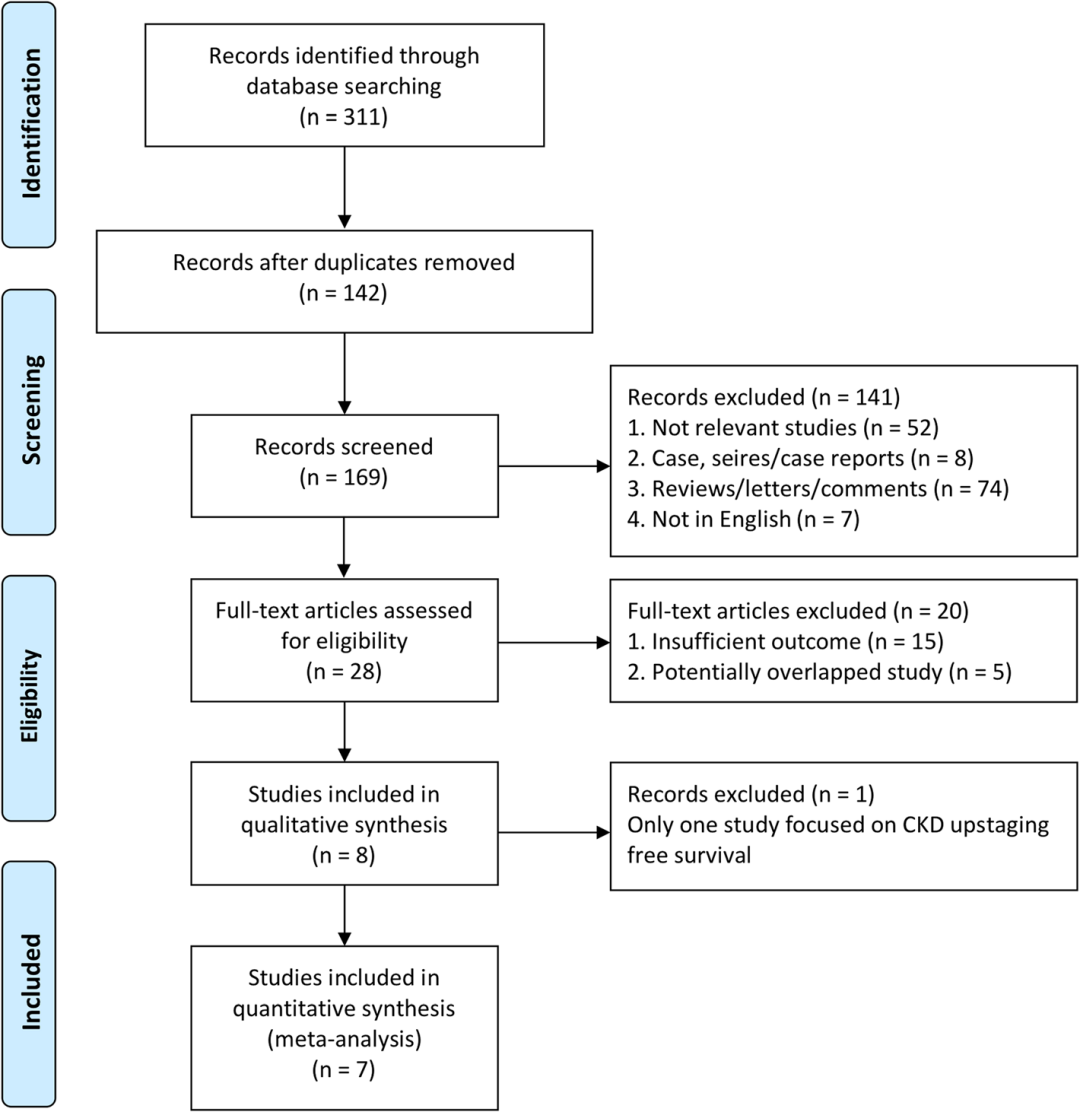
Flowchart of study selection

A total of 311 potentially relevant studies were identified. 142 duplicated publications were excluded through literature manager software (Endnote). According to the titles and abstracts, 141 were precluded: 52 were irrelevant studies, eight were case/series/case reports, 74 were letters/reviews/comments, seven were not in English or Chinese. After reviewed in depth, 15 publications were excluded due to inadequate outcome and five was excluded due to potentially overlapping populations. Additionally, one study was excluded because only this study focused on chronic kidney disease upstaging free survival. Finally, seven studies were included in the present meta-analysis [14–20] (Fig. 1). Table 1 summarized the study design characteristics [14–20].

**Table 1:** Study design characteristics of included studies in meta-analysis

Study	Year	Region/Country	Study period	Study design	Database	No. of patients	Intervention	Control	Inclusion criteria	Exclusion criteria	Outcome	Quality score
Alam R et al [14]	2018	USA	2009-NA	Prospective cohort	Delayed Intervention and Surveillance for Small Renal Masses Registry	597	PN, TA (CA, RFA)	AS	Age > 18, cT1a renal mass	History of RCC, familial RCC syndrome, or suspicion of a second malignancy metastasis	CSS, OS	9
Miller BL et al [15]	2018	USA	2003–2016	Retrospective	Single center data	135	PN, TA	AS	Histological diagnosis of oncocytoma or chRCC (cT1–2)	NA	CSS, OS	7
Tang DH et al [16]	2017	USA	2000–2013	Retrospective	Single center data	62	PN	AS	Age 80–89, cT1–2 renal mass	NA	CSS, OS	7
Larcher A et al [17]	2015	USA	2000–2009	Retrospective	SEER-Medicare-linked database	1860	TA	AS	T1aN0M0, unilateral RCC	RCC diagnosed only on death	CSS	9
Patel HD et al [18]	2015	USA	1995–2007	Retrospective	SEER-Medicare-linked database	2603	PN	AS	cT1aN0M0 renal cortical tumor	cT3–4, N1–2, M1, unknown classification, urothelial carcinoma, noncortical renal tumors, multiple procedures, bilateral tumors, previous diagnosis of another cancer, undergone TA	CSS, OS, CVSS	8
Patel N et al [19]	2012	UK	2005–2010	Retrospective	Cancer Research Uro-Oncology Database	161	PN	AS	cT1aN0M0 renal mass	NA	CSS, OS	7
Lane BR et al [20]	2010	USA	2000–2006	Retrospective	Single center data	391	open/laparoscopic PN, TA (CA, RFA)	AS	Age > 75, cT1 renal tumor	Not suspicious renal tumor, PN for other reason	CSS, OS, CVSS	8

*AS* active surveillance, *CA* cryoablation, *CSS* cancer-specific survival, *CVSS* cadiovascular-specific survival, *NA* not available, *OS* overall survival, *PN* partial nephrectomy, *RCC* renal cell carcinoma, *RFA* radiofrequency ablation, *SEER* Surveillance, Epidemiology, and End Results, *TA* thermal ablation

The seven studies contained 5809 patients with renal mass, including 3112 patients who received NSI and 2697 patients in AS group. Of the seven studies, all seven studies were conducted to compare the CSS between NSI group and AS group [14–20], six studies containing 3949 patients compared the OS [14–16, 18–20], and two studies containing 2994 patients compared CVSS [18, 20].

Study qualities were assessed based on the modified Newcastle-Ottawa scale. All the seven studies reached stars ranging from seven to nine and were identified as moderate to high quality (Table 1). The baseline characteristics of included studies (number of patients received NSI or AS, gender, median age, tumor size and follow-up duration) were shown in Table 2.

**Table 2:** Baseline characteristics of included studies in meta-analysis

Study detail	No. of patients, type	Sex, Male/Female	Median age (years)	Median tumor size (centimeter)	Median follow-up duration (months)
Alam R et al 2018 [14]	PN 231	145/86	61.3	2.4	
	TA 27	13/14	71.8	2.1	36.0 (Total)
	AS 339	190/149	70.6	1.8	
	AS 1978	NA	NA	2.8	> 42.2
Miller BL et al 2018 [15]	PN 31	NA	Oncocytoma 69.5 chRCC 57.0	NA	
	TA 14	NA		NA	39.9 (Total)
	AS 90	NA		NA	
Tang DH et al 2017 [16]	PN 31	24/7	81.0	3.2	51.0 (Total)
	AS 31	18/13	83.0	2.7	
Larcher A et al 2015 [17]	TA 553	308/245	77.0	2.7	30.0 (Total)
	AS 1307	778/529	78.0	2.8	
Patel HD et al 2015 [18]	PN 1849	1070/779	65–69 589(31.5%) 70–74 569(27.5%) 75–79 463(25.2%) 80–84 191(19.5%) ≥85 37(8.9%)	< 2 cm 454(41.2%) 2- < 3 cm 776(31.8%) 3- ≤ 4 cm 614 (17.0%)	56.0 (Total)
	AS 754	399/355	65–69 589(31.5%) 70–74 569(27.5%) 75–79 463(25.2%) 80–84 191(19.5%) ≥85 37(8.9%)	< 2 cm 134(12.2%) 2- < 3 cm 258(10.6%) 3- ≤ 4 cm 361(10.0%)	
Patel N et al 2012 [19]	PN 90	NA	58.9	2.69	33.0
	AS 71	52/19	71.9	2.2	34.0
Lane BR et al 2010 [20]	NSI 286	200/86	78.0	3.0	46.8 (Total)
	AS 105	58/47	81.0	2.3	

*AS* active surveillance, *chRCC* chromophobe renal cell carcinoma, *NA* not available, *NSI* nephron-sparing intervention, *PN* partial nephrectomy, *TA* thermal ablation

### Meta-analysis results

#### Cancer-specific survival

**Fig. 2:**
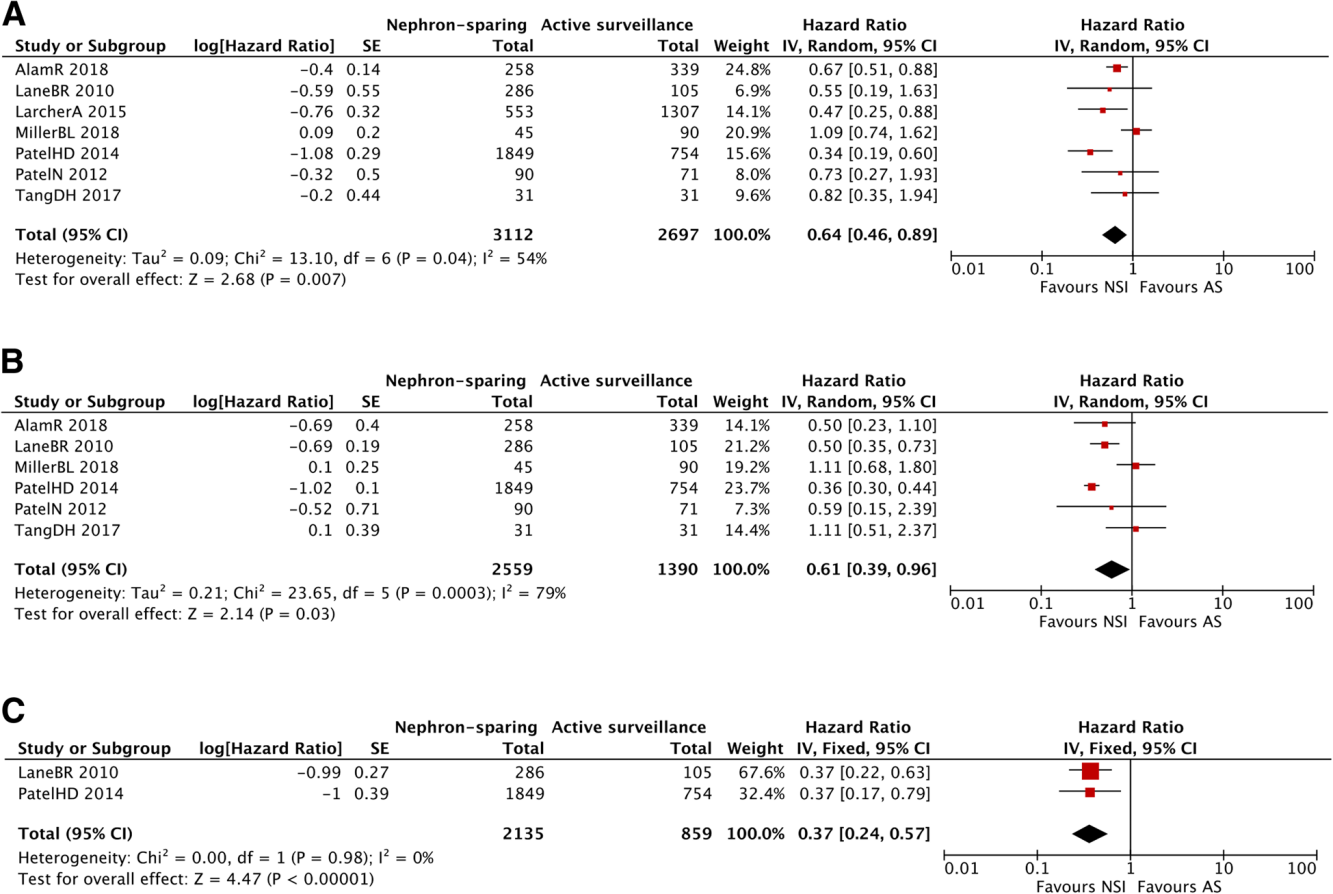
Forest plot comparing cancer-specific survival in patients receiving nephron-sparing intervention versus active surveillance. **a** cancer-specific survival; **b** overall survival; **c** cardiovascular-specific survival

Seven studies addressed to CSS, of which two were studies of the SEER dataset [17, 18]. The combined HR of these studies revealed that NSI was associated with a statistically significant CSS benefit compared with AS for patients with renal masses (HR = 0.64, 95%CI: 0.46–0.89, *P* < 0.001) with apparent inter-study heterogeneity (*I*^*2*^ = 54%, *Chi*^*2*^ = 13.10, *P* = 0.04) (Fig. 2a). To explore the source of the heterogeneity, meta-regression analysis and subgroup analysis were conducted by quality of the study, publication year, patient sample, type of intervention, and tumor size. The results showed that patient sample (*P* = 0.030) and tumor size (*P* = 0.046) might have significant association with the heterogeneity, while other factors did not (Table 3). Both population-based SEER and non-SEER studies demonstrated a CSS advantage of NSI over AS (HR = 0.39, 95%CI: 0.26–0.60, *P* < 0.001; HR = 0.78, 95%CI: 0.63–0.96, *P* = 0.02, respectively). Furthermore, subgroup analysis showed that both PN and TA were associated with better CSS when compared with AS (HR = 0.67, 95%CI: 0.49–0.90, *P* = 0.008; HR = 0.68, 95%CI: 0.52–0.89, *P* = 0.005, respectively). When confined to studies with T1a renal tumor, patients undergoing NSI showed a better CSS than those in AS (HR = 0.68, 95%CI: 0.56–0.82, *P* < 0.001).

**Table 3:** Subgroup analysis of CSS and OS by quality of the study, publication year, patient sample, number or type of intervention, and tumor size

Subgroup/co-variant	Coefficient	s.e.	*P* value	HR (95%CI); *P* value
CSS				
Quality	− 0.227	0.1580	*0.195*	
Publication year	0.598	0.2929	*0.096*	
≤ 2015				0.44 (0.31–0.64); *< 0.001*
> 2015				0.76 (0.65–0.89); *0.03*
Patient sample	−0.688	0.2539	*0.030*	
Cancer registry or single center				0.78 (0.63–0.96); *0.02*
Population based				0.39 (0.26–0.60); *< 0.001*
Type of intervention	−0.014	0.2628	*0.958*	
PN				0.67 (0.49–0.90); *0.008*
TA				0.68 (0.52–0.89); *0.005*
Tumor size	−0.567	0.2342	*0.046*	
T1a				0.68 (0.56–0.82); *< 0.001*
Not only T1a				0.70 (0.36–1.38); *0.31*
OS				
Quality	−0.446	0.1986	*0.066*	
Publication year	0.560	0.3845	*0.196*	
≤ 2015				0.39 (0.33–0.46); *< 0.001*
> 2015				0.93 (0.65–1.34); *0.70*
Patient sample	−0.658	0.4973	*0.234*	
Cancer registry or single center				0.68 (0.53–0.88); *0.004*
Population based				0.36 (0.30–0.44); *< 0.001*
Intervention	0.625	0.2489	*0.049*	
PN				0.40 (0.34–0.47); *< 0.001*
TA				1.05 (0.62–1.80); *0.85*
Tumor size	−0.681	0.3091	*0.070*	
T1a				0.43 (0.36–0.51); *< 0.001*
Not only T1a				0.58 (0.42–0.82); *0.002*

*CI* confidence interval, *CSS* cancer-specific survival, *OS* overall survival, *PN* partial nephrectomy, *TA* thermal ablation

#### Overall survival

Six studies including one SEER study [18] reported data for OS, and there was significant heterogeneity (*I*^*2*^ = 79%, *Chi*^*2*^ = 23.65, *P* = 0.0003); thus, the random-effects model was utilized. The combined HR of these studies revealed that NSI was associated with better OS (HR = 0.46, 95%CI: 0.39–0.53, *P* < 0.001) (Fig. 2b). OS subgroup analysis revealed that PN was responsible for weighting the pooled estimate for a better OS (HR = 0.40, 95%CI: 0.34–0.47, *P* < 0.0001), while patients may not benefit from TA in OS (Table 3). When confined to studies with T1a renal tumor, the pooled results (HR = 0.43, 95%CI: 0.36–0.51, *P* < 0.001) indicated that NSI had a positive impact on the OS for these patients.

#### Cadiovascular-specific survival

No obvious heterogeneity was observed in the two studies focusing on CVSS (*I*^*2*^ = 0%, *Chi*^*2*^ = 0, *P* = 0.98), thus the fixed-effects model was applied. The pooled HR for CVSS was 0.37 (95%CI: 0.24–0.57, *P* < 0.001), favoring NSI for patients with renal masses (Fig. 2c).

### Publication bias

**Fig. 3:**
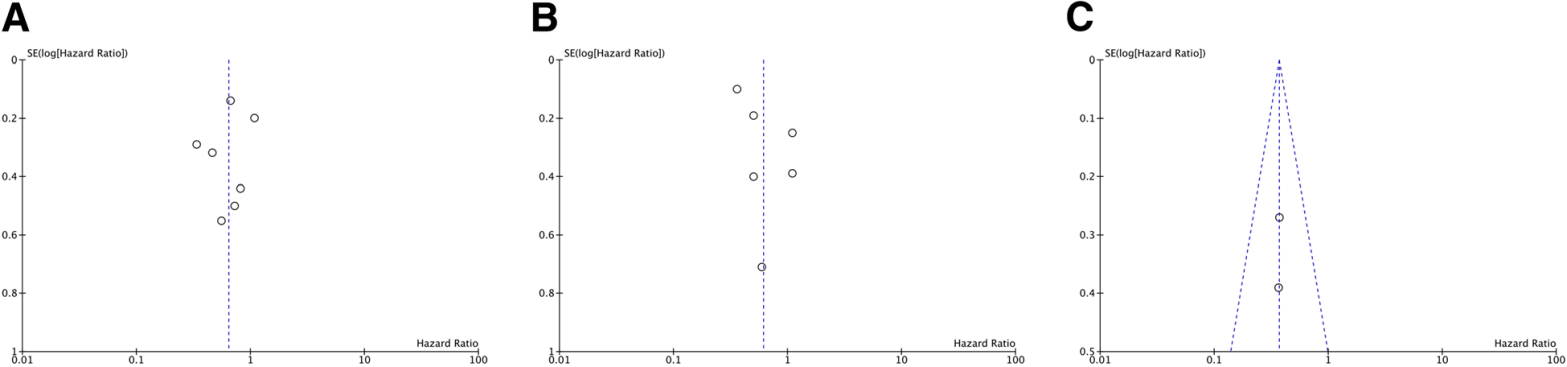
Funnel plot for the evaluation of potential publication bias. **a** cancer-specific survival; **b** overall survival; **c** cardiovascular-specific survival

Funnel plots were conducted to detect the publication bias. As shown in Fig. 3, no obvious publication bias was found in our meta-analysis of included studies. And the Egger’s test demonstrated that there was no publication bias for CSS (P_egger_ = 0.649, intercept − 0.55 with 95%CI − 3.50 to 2.40) and OS (P_egger_ = 0.129, intercept 2.49 with 95%CI − 1.13 to 6.10).

## Discussion

The option for localized renal tumors has experienced extraordinary change with the advent of minimally invasive and nephron-sparing interventions. AS represents the least invasive management and has been exploited selectively among patients with limited life expectancy for renal tumors with low malignant potential. The effectiveness of PN, TA, and AS for patients with renal tumors has attracted extensive attention and been widely debated; however, the strength of evidence remains low to moderate, and the lack of data regarding AS rendered analysis largely insufficient to draw conclusions [21]. Thus, we reviewed the studies and the principal findings of our study are relevant to the management options for node-negative non-metastatic renal tumor.

To summarize, NSI was associated with better OS and CSS when compared with AS, and was even better when confined to T1a renal tumor. Both PN and TA showed an apparently better CSS when compared with AS. According to Lane BR *et al* [20], although no difference was observed in cancer-specific mortality between AS and NSI (PN and TA), there were apparent differences in characteristics of patients and tumors between the comparing groups, and the proportion of malignancy in AS might be diluted to an unknown extent. Nevertheless, a study by Larcher A *et al* [17] mentioned that after adjustment for other cause mortality and various characteristics of patients and tumors, TA seemed to be associated with a protective effect on cancer specific mortality when compared with observation, and resulted in acceptable peri-operative morbidity [21]. We also noted that PN demonstrated better OS than AS, while TA did not. It was hypothesized that patients who underwent TA might be poor surgical candidates, as the current American Urological Association [22], European Association for Urology [23], and National Comprehensive Cancer Network guidelines [24] recommend. However, these recommendations are chiefly based on older TA data with higher local tumor recurrence rates and incomplete ablation rates [22–24]. Thus, with the development of improved ablative technologies, TA and PN could achieve comparable CSS. Additionally, relative long follow-up duration affirms the safety of AS compared to NSI among the selected population, who are usually older and have increased comorbidities. These factors probably explain the lower OS rate among AS patients and suggest that OS in AS group might be driven primarily by comorbidities [18, 25, 26]. Patel HD *et al* [18] stressed that patients who underwent nonsurgical management at the population-level in the SEER studies might not be representative of patients selected for AS in contemporary criteria. Besides, underestimating tumor stage might also contribute to the cancer-specific mortality in these nonsurgical management group. The elderly might also have a more aggressive tumor with the hypothesis that relative immunodeficiency associated with aging might facilitate rapid growth of RCC [27].

Interestingly, NSI was obviously associated with better CVSS, suggesting that cancer may be not the main cause of death for most patients undergoing AS. It is our hypothesis that the selection bias might contribute to this result. The AS group represents an extreme condition where patients did not experience intervention (especially surgery) for some reasons, but the most likely are age, comorbidities, tumor characteristics, and patient preference. A population-based competing risk analysis by Hollingsworth *et al* [28] demonstrated that the competing-cause mortality for elderly patients (aged > 70 years) was 28%. In such cases, the surgeon and the patient would omit intervention and choose AS, which aims to reduce potential overtreatment without conceding oncologic outcomes [29] and may result in worse CVSS in the AS group. Therefore, patients at high cardiovascular risk are very reasonable candidates for AS [18].

To the best of our knowledge, this is the first time that a comprehensive systematic review and meta-analysis has compared the effectiveness between NSI (PN and TA included) and AS for patients with renal masses; nevertheless, this meta-analysis has a few limitations to be noted. First, the sources of the publications were limited, and we failed to retrieve unpublished studies or studies written in other language, therefore potentially introducing inevitable publication biases. Second, only two studies in this research was prospective cohort studies. Despite the high quality (> 6 stars) of the seven studies, intrinsic bias might have rendered the results less trustworthy. Third, we failed to compare the survival according to age, Charlson comorbidity index and histology due to lack of information. Increased age and Charlson comorbidity index are associated with a higher hazard of all-cause mortality [17, 18, 30]. Fourth, there were only two studies compare CVSS between NSI and AS even after a systematic literature search, and the risk of random error might have inevitably increased; thus, larger prospective cohort studies are needed to further verify our findings. Furthermore, studies of TA and AS may include patients with benign tumors and may overestimate the efficacy of these management options. Improved diagnostics and judicious use of renal mass biopsy may improve the understanding of tumor biology. Finally, the outcomes were influenced by the quality of selected studies and the reporting bias that papers with significant outcomes were easier to be published than those with null or nonsignificant results might be unavoidable [31].

## Conclusions

In conclusion, notwithstanding the limitations of this systematic review and meta-analysis, it seems that NSI is associated with better OS, CSS and CVSS when compared with AS for patients with renal masses. Further better prospective cohort studies with matched groups based on comorbidities and age are needed to make definitive statements about these different treatment methods.

## Data Availability

Not applicable.

## Abbreviations

AS: Active surveillance
CI: Confidence interval
CSS: Cancer-specific survival
CVSS: Cardiovascular-specific survival
HR: Hazard ratio
NSI: Nephron-sparing intervention
OS: Overall survival
PN: Partial nephrectomy
RCC: Renal cell carcinoma
TA: Thermal ablation
